# Supplementary material for: Psoriatic Resolved Skin Epidermal Keratinocytes Retain Disease-Residual Transcriptomic and Epigenomic Profiles
Source: Int J Mol Sci. 2023 Feb 25;24(5):4556. doi: 10.3390/ijms24054556 (PMC10002496; doi:10.3390/ijms24054556)
Supplement: Supplementary file 1 [file ijms-24-04556-s001.zip › ijms-2143985-supplementary.pdf]

Supplementary Materials

(a)

(b)

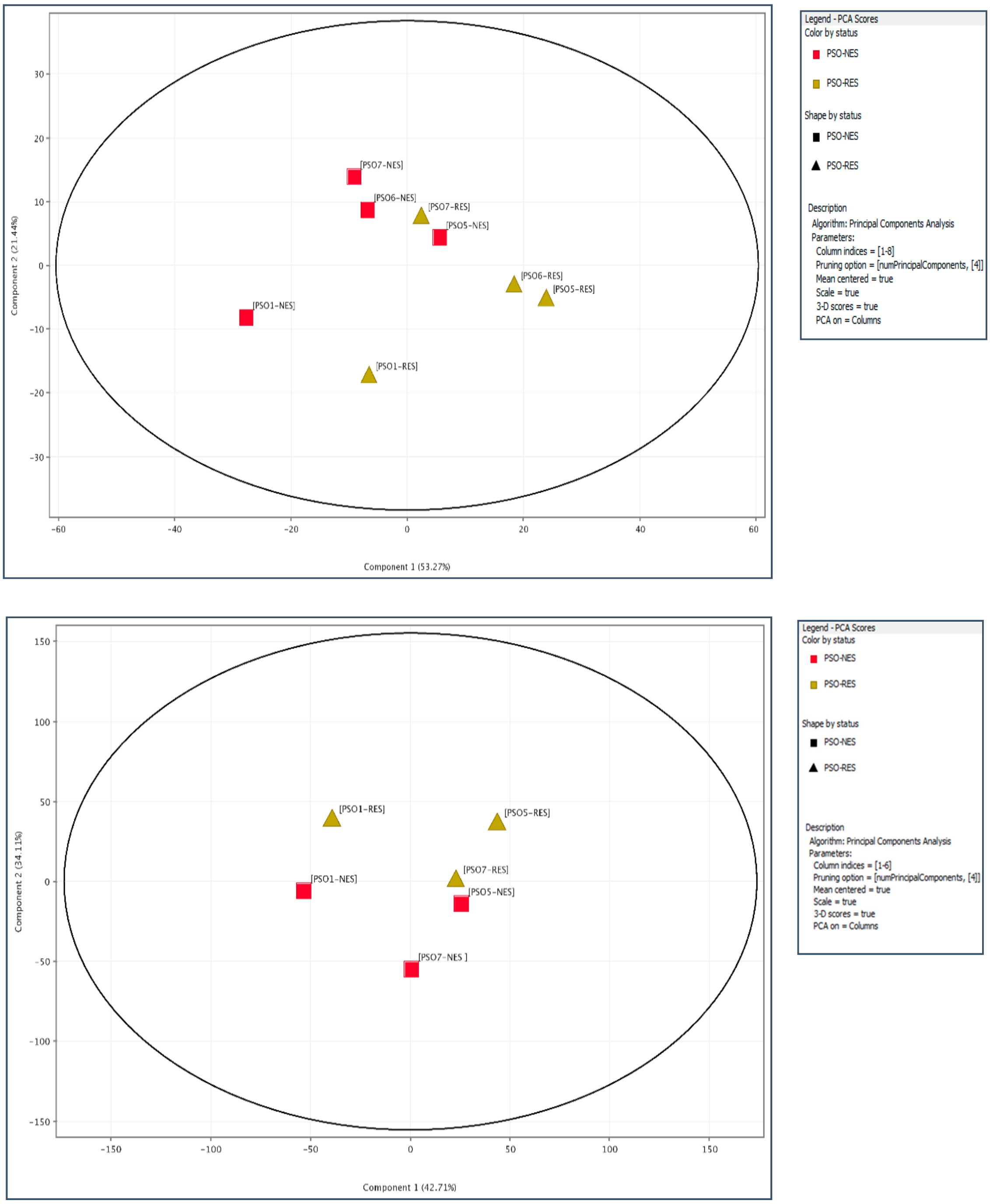

Figure S1. Principal component analysis (PCA). PCA plots of (**a**) epidermal and (**b**) dermal paired never-lesional and resolved samples. PCA was performed on resolved *vs.* never-lesional DEGs. Each point represents an experimental sample; colors and shapes indicate different types of samples. Abbreviations: RES, resolved skin; NES, never-lesional skin; DEG, differentially expressed genes.

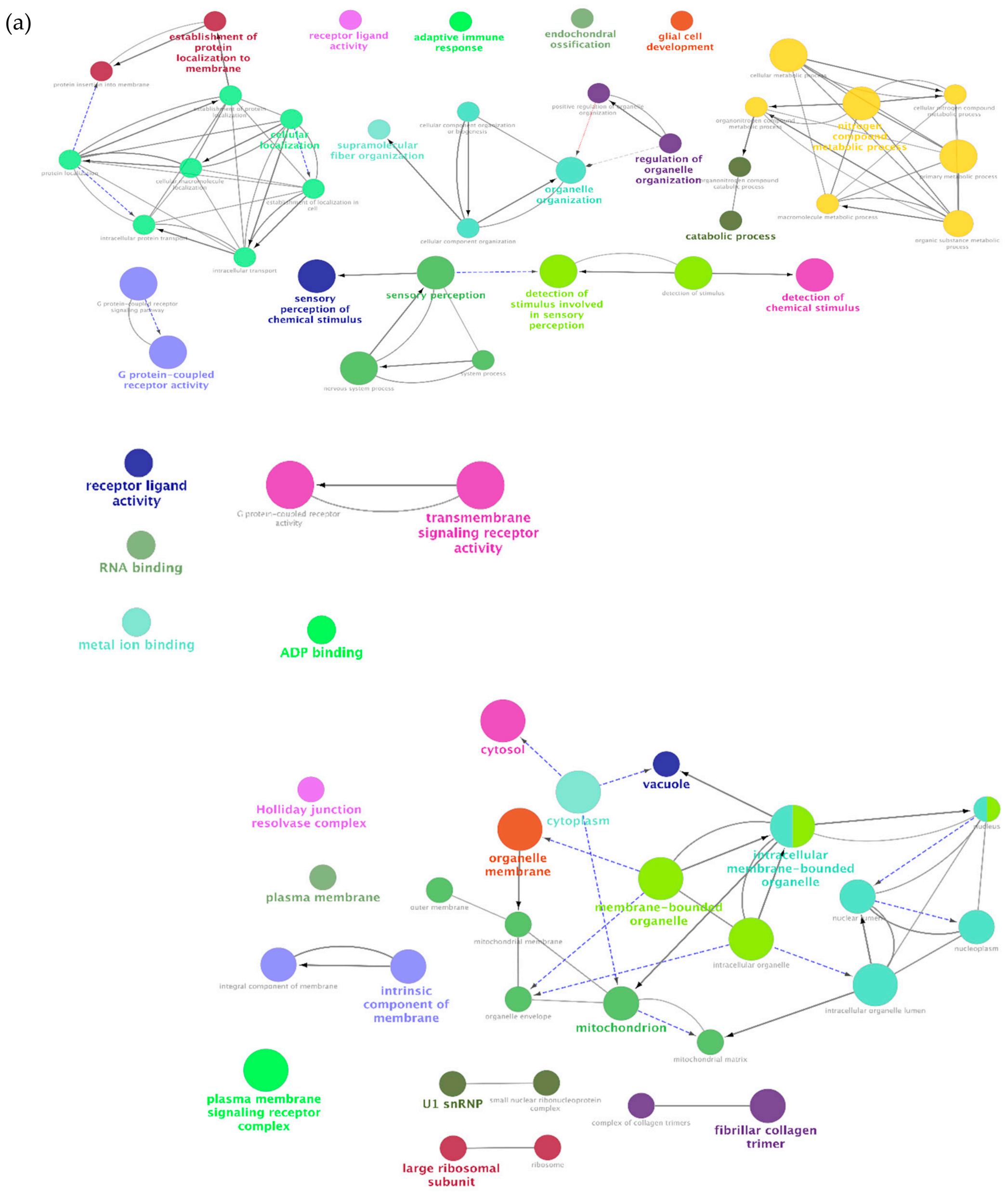

Figure S2. GO enrichment analysis of resolved *vs.* never-lesional DEGs in dermal compartments. Enrichment shows only significant (**a**) BPs, (**b**) MFs, and (**c**) CCs with *p* <0.05. Node color represents the specific functional class, biological processes, and cellular components involved in the enrichment analysis of the identified DEGs. Bold fonts indicate the major BPs, MFs, or CCs that define the names of each group. Abbreviations: DEG, differentially expressed genes; BPs, biological processes; MFs, molecular functions; CCs, cellular components.

Table S1. List of the 102 overlapped genes between resolved *vs.* never-lesional DEGs and lesional *vs.* healthy DEGs in the epidermis.

Number	*Gene symbol*	*Gene ID*	*P-value*	*Regulation*	*FC*	*Description*
**1**	CACNA2D1	ENSG00000153956	0.0029	down	-2.49	calcium voltage-gated channel auxiliary subunit alpha2delta 1
**2**	ODF3L1	ENSG00000182950	0.0290	down	-2.05	outer dense fiber of sperm tails 3 like 1
**3**	WNT2	ENSG00000105989	0.0430	down	-2.03	Wnt family member 2
**4**	LIF	ENSG00000128342	0.0372	down	-1.88	leukemia inhibitory factor
**5**	TPPP	ENSG00000171368	0.0014	down	-1.83	tubulin polymerization promoting protein
**6**	LRRC8C	ENSG00000171488	0.0100	down	-1.69	leucine rich repeat containing 8 family member C
**7**	ANKRD33B	ENSG00000164236	0.0040	down	-1.66	ankyrin repeat domain 33B
**8**	KLF11	ENSG00000172059	0.0349	down	-1.63	Kruppel like factor 11
**9**	MLXIP	ENSG00000175727	0.0133	down	-1.57	MLX interacting protein
**10**	PAMR1	ENSG00000149090	0.0500	down	-1.57	peptidase domain containing associated with muscle regeneration 1
**11**	CERS6	ENSG00000172292	0.0155	down	-1.56	ceramide synthase 6
**12**	ATP2B1	ENSG00000070961	0.0383	down	-1.50	ATPase plasma membrane Ca2+ transporting 1
**13**	HSF2	ENSG00000025156	0.0101	down	-1.50	heat shock transcription factor 2
**14**	ABCA3	ENSG00000167972	0.0074	down	-1.48	ATP binding cassette subfamily A member 3
**15**	CNTNAP3	ENSG00000106714	0.0304	down	-1.47	contactin associated protein-like 3
**16**	RHOBTB3	ENSG00000164292	0.0378	down	-1.45	Rho related BTB domain containing 3
**17**	CDON	ENSG00000064309	0.0271	down	-1.45	cell adhesion associated, oncogene regulated
**18**	DUSP10	ENSG00000143507	0.0085	down	-1.44	dual specificity phosphatase 10
**19**	CLDN8	ENSG00000156284	0.0171	down	-1.44	claudin 8
**20**	ZNF614	ENSG00000142556	0.0108	down	-1.43	zinc finger protein 614
**21**	EDIL3	ENSG00000164176	0.0090	down	-1.43	EGF like repeats and discoidin domains 3
**22**	ESPN	ENSG00000187017	0.0245	down	-1.42	espin
**23**	RASAL2	ENSG00000075391	0.0326	down	-1.42	RAS protein activator like 2
**24**	ZXDB	ENSG00000198455	0.0343	down	-1.41	zinc finger, X-linked, duplicated B
**25**	N4BP2L1	ENSG00000139597	0.0185	down	-1.40	NEDD4 binding protein 2 like 1
**26**	RNF217	ENSG00000146373	0.0019	down	-1.40	ring finger protein 217
**27**	EEF2K	ENSG00000103319	0.0131	down	-1.39	eukaryotic elongation factor 2 kinase
**28**	FCER1A	ENSG00000179639	0.0003	down	-1.38	Fc fragment of IgE receptor Ia
**29**	RICTOR	ENSG00000164327	0.0260	down	-1.37	RPTOR independent companion of MTOR complex 2
**30**	ZC3H12A	ENSG00000163874	0.0224	down	-1.37	zinc finger CCCH-type containing 12A
**31**	GATM	ENSG00000171766	0.0024	down	-1.36	glycine amidinotransferase
**32**	HIVEP1	ENSG00000095951	0.0471	down	-1.35	human immunodeficiency virus type I enhancer binding protein 1
**33**	AAMDC	ENSG00000087884	0.0023	down	-1.35	adipogenesis associated Mth938 domain containing
**34**	TRAF6	ENSG00000175104	0.0401	down	-1.34	TNF receptor associated factor 6
**35**	EFHC2	ENSG00000183690	0.0191	down	-1.33	EF-hand domain containing 2
**36**	DIP2B	ENSG00000066084	0.0036	down	-1.33	disco interacting protein 2 homolog B
**37**	SIK1	ENSG00000142178	0.0144	down	-1.32	salt inducible kinase 1
**38**	TNFRSF19	ENSG00000127863	0.0208	down	-1.32	TNF receptor superfamily member 19
**39**	FGD4	ENSG00000139132	0.0179	down	-1.31	FYVE, RhoGEF and PH domain containing 4
**40**	UBN2	ENSG00000157741	0.0366	down	-1.31	ubinuclein 2
**41**	PHC3	ENSG00000173889	0.0424	down	-1.31	polyhomeotic homolog 3
**42**	CLDN1	ENSG00000163347	0.0165	down	-1.31	claudin 1
**43**	PPTC7	ENSG00000196850	0.0017	down	-1.30	PTC7 protein phosphatase homolog
**44**	DZIP1L	ENSG00000158163	0.0243	down	-1.30	DAZ interacting zinc finger protein 1 like
**45**	ANO1	ENSG00000131620	0.0170	down	-1.30	anoctamin 1
**46**	DENND4C	ENSG00000137145	0.0159	down	-1.29	DENN domain containing 4C
**47**	MAP3K2	ENSG00000169967	0.0164	down	-1.29	mitogen-activated protein kinase kinase kinase 2
**48**	GPCPD1	ENSG00000125772	0.0071	down	-1.29	glycerophosphocholine phosphodiesterase 1
**49**	KLF13	ENSG00000169926	0.0214	down	-1.28	Kruppel like factor 13
**50**	ARHGAP42	ENSG00000165895	0.0069	down	-1.28	Rho GTPase activating protein 42

**51**	PHYHD1	ENSG00000175287	0.0348	down	-1.26	phytanoyl-CoA dioxygenase domain containing 1
**52**	SPATA18	ENSG00000163071	0.0154	down	-1.26	spermatogenesis associated 18
**53**	BACH1	ENSG00000156273	0.0094	down	-1.26	BTB domain and CNC homolog 1
**54**	ATG14	ENSG00000126775	0.0185	down	-1.26	autophagy related 14
**55**	CEP68	ENSG00000011523	0.0305	down	-1.25	centrosomal protein 68
**56**	TUBGCP3	ENSG00000126216	0.0244	down	-1.25	tubulin gamma complex associated protein 3
**57**	FZD1	ENSG00000157240	0.0303	down	-1.25	frizzled class receptor 1
**58**	RC3H1	ENSG00000135870	0.0082	down	-1.25	ring finger and CCCH-type domains 1
**59**	NIPA1	ENSG00000170113	0.0310	down	-1.24	non imprinted in Prader-Willi/Angelman syndrome 1
**60**	PAN3	ENSG00000152520	0.0052	down	-1.24	PAN3 poly(A) specific ribonuclease subunit
**61**	SVIP	ENSG00000198168	0.0349	down	-1.22	small VCP interacting protein
**62**	MBNL1	ENSG00000152601	0.0129	down	-1.22	muscleblind like splicing regulator 1
**63**	ZFP36L2	ENSG00000152518	0.0286	down	-1.21	ZFP36 ring finger protein like 2
**64**	TBL1XR1	ENSG00000177565	0.0258	down	-1.21	transducin beta like 1 X-linked receptor 1
**65**	C1GALT1	ENSG00000106392	0.0215	down	-1.21	core 1 synthase, glycoprotein-N-acetylgalactosamine 3-beta-galactosyltransferase 1
**66**	IRF6	ENSG00000117595	0.0296	down	-1.20	interferon regulatory factor 6
**67**	LNPEP	ENSG00000113441	0.0403	down	-1.19	leucyl and cystinyl aminopeptidase
**68**	AKR1B10	ENSG00000198074	0.0387	up	2.58	aldo-keto reductase family 1 member B10
**69**	FABP5	ENSG00000164687	0.0198	up	2.09	fatty acid binding protein 5
**70**	PYDC1	ENSG00000169900	0.0150	up	2.04	pyrin domain containing 1
**71**	WNT5A	ENSG00000114251	0.0132	up	1.92	Wnt family member 5A
**72**	TMEM52	ENSG00000178821	0.0177	up	1.62	transmembrane protein 52
**73**	SCD	ENSG00000099194	0.0127	up	1.53	stearoyl-CoA desaturase
**74**	TREX2	ENSG00000183479	0.0397	up	1.48	three prime repair exonuclease 2
**75**	ROM1	ENSG00000149489	0.0256	up	1.47	retinal outer segment membrane protein 1
**76**	TK1	ENSG00000167900	0.0441	up	1.47	thymidine kinase 1
**77**	PON3	ENSG00000105852	0.0006	up	1.47	paraoxonase 3
**78**	GADD45G	ENSG00000130222	0.0248	up	1.46	growth arrest and DNA damage inducible gamma
**79**	CTSB	ENSG00000164733	0.0235	up	1.45	cathepsin B
**80**	TMEM64	ENSG00000180694	0.0373	up	1.44	transmembrane protein 64
**81**	TMEM14A	ENSG00000096092	0.0085	up	1.43	transmembrane protein 14A
**82**	MYO1B	ENSG00000128641	0.0116	up	1.41	myosin IB
**83**	WNT10A	ENSG00000135925	0.0262	up	1.40	Wnt family member 10A
**84**	RASA4	ENSG00000105808	0.0411	up	1.40	RAS p21 protein activator 4
**85**	ZNF503	ENSG00000165655	0.0010	up	1.37	zinc finger protein 503
**86**	GALNT6	ENSG00000139629	0.0177	up	1.36	polypeptide N-acetylgalactosaminyltransferase 6
**87**	TNNT2	ENSG00000118194	0.0274	up	1.35	troponin T2, cardiac type
**88**	STK39	ENSG00000198648	0.0300	up	1.35	serine/threonine kinase 39
**89**	SLX1A	ENSG00000132207	0.0085	up	1.35	SLX1 homolog A, structure-specific endonuclease subunit
**90**	CRABP2	ENSG00000143320	0.0207	up	1.34	cellular retinoic acid binding protein 2
**91**	DTYMK	ENSG00000168393	0.0152	up	1.34	deoxythymidylate kinase
**92**	PLBD1	ENSG00000121316	0.0194	up	1.34	phospholipase B domain containing 1
**93**	VASN	ENSG00000168140	0.0234	up	1.33	vasorin
**94**	PUDP	ENSG00000130021	0.0446	up	1.32	pseudouridine 5'-phosphatase
**95**	AKR1B1	ENSG00000085662	0.0092	up	1.30	aldo-keto reductase family 1 member B
**96**	ESRRA	ENSG00000173153	0.0385	up	1.30	estrogen related receptor alpha
**97**	BEX3	ENSG00000166681	0.0380	up	1.28	brain expressed X-linked 3
**98**	SRXN1	ENSG00000271303	0.0201	up	1.28	sulfiredoxin 1
**99**	PDLIM4	ENSG00000131435	0.0434	up	1.26	PDZ and LIM domain 4
**100**	POLL	ENSG00000166169	0.0259	up	1.26	DNA polymerase lambda
**101**	CDKN1C	ENSG00000129757	0.0401	up	1.22	cyclin dependent kinase inhibitor 1C
**102**	PON2	ENSG00000105854	0.0194	up	1.21	paraoxonase 2

Table S2. GO enrichment analysis of the 102 overlapped genes between resolved *vs.* never-lesional DEGs and lesional *vs.* healthy DEGs in the epidermis. We performed GO analysis on 102 overlapped genes. The results revealed 54 significant BPs (P < 0.001 & FDR < 0.2). Abbreviations: GO, gene ontology; DEGs, differentially expressed genes; BP, biological processes.

Number	*GO Term*	*Description*	*P-value*	*FDR q-value*	*Enrichment*
**1**	GO:0051716	cellular response to stimulus	0.00000419	0.0644	2.26
**2**	GO:0071560	cellular response to transforming growth factor beta stimulus	0.00000613	0.0471	19.19
**3**	GO:0071559	response to transforming growth factor beta	0.0000127	0.0649	16.59
**4**	GO:0048518	positive regulation of biological process	0.0000147	0.0565	1.67
**5**	GO:1904953	Wnt signaling pathway involved in midbrain dopaminergic neuron differentiation	0.0000151	0.0465	58.72
**6**	GO:0042221	response to chemical	0.0000164	0.042	2.24
**7**	GO:0070887	cellular response to chemical stimulus	0.0000165	0.0362	2.67
**8**	GO:0071310	cellular response to organic substance	0.0000262	0.0504	2.87
**9**	GO:0071495	cellular response to endogenous stimulus	0.0000434	0.0742	3.73
**10**	GO:0045598	regulation of fat cell differentiation	0.0000546	0.084	9.03
**11**	GO:0048856	anatomical structure development	0.0000726	0.101	1.97
**12**	GO:0046125	pyrimidine deoxyribonucleoside metabolic process	0.0000773	0.099	130.48
**13**	GO:0046104	thymidine metabolic process	0.0000773	0.0914	130.48
**14**	GO:0006796	phosphate-containing compound metabolic process	0.0000824	0.0905	2.34
**15**	GO:0061158	3'-UTR-mediated mRNA destabilization	0.0000835	0.0856	34.54
**16**	GO:0006793	phosphorus metabolic process	0.000102	0.0979	2.31
**17**	GO:0032872	regulation of stress-activated MAPK cascade	0.000121	0.11	6.34
**18**	GO:0070302	regulation of stress-activated protein kinase signaling cascade	0.000132	0.113	6.26
**19**	GO:0032874	positive regulation of stress-activated MAPK cascade	0.000139	0.113	7.63
**20**	GO:0070304	positive regulation of stress-activated protein kinase signaling cascade	0.00015	0.115	7.53
**21**	GO:0009893	positive regulation of metabolic process	0.000227	0.166	1.83
**22**	GO:0046328	regulation of JNK cascade	0.000231	0.161	6.95
**23**	GO:0048522	positive regulation of cellular process	0.000313	0.209	1.61
**24**	GO:0051239	regulation of multicellular organismal process	0.000341	0.219	1.87
**25**	GO:0071363	cellular response to growth factor stimulus	0.000351	0.216	5.33
**26**	GO:0009120	deoxyribonucleoside metabolic process	0.000383	0.226	65.24
**27**	GO:0071470	cellular response to osmotic stress	0.000386	0.22	20.97
**28**	GO:0006970	response to osmotic stress	0.000406	0.223	11.51
**29**	GO:0060070	canonical Wnt signaling pathway	0.000406	0.215	11.51
**30**	GO:0016310	phosphorylation	0.00041	0.21	2.69
**31**	GO:0046330	positive regulation of JNK cascade	0.000438	0.217	7.89
**32**	GO:0006468	protein phosphorylation	0.000448	0.215	2.94
**33**	GO:1902105	regulation of leukocyte differentiation	0.000493	0.23	5.04
**34**	GO:0009719	response to endogenous stimulus	0.000497	0.225	2.77
**35**	GO:0042538	hyperosmotic salinity response	0.000534	0.234	55.92
**36**	GO:0051173	positive regulation of nitrogen compound metabolic process	0.000535	0.228	1.85
**37**	GO:0010033	response to organic substance	0.000535	0.222	2.11
**38**	GO:0061157	mRNA destabilization	0.000576	0.233	18.35
**39**	GO:0010604	positive regulation of macromolecule metabolic process	0.000585	0.23	1.81
**40**	GO:0050896	response to stimulus	0.000594	0.228	1.55
**41**	GO:0051254	positive regulation of RNA metabolic process	0.0006	0.225	2.26
**42**	GO:0070848	response to growth factor	0.000664	0.243	4.79
**43**	GO:0003056	regulation of vascular smooth muscle contraction	0.000709	0.254	48.93
**44**	GO:0120161	regulation of cold-induced thermogenesis	0.000737	0.257	7.04
**45**	GO:1901701	cellular response to oxygen-containing compound	0.000741	0.253	2.95
**46**	GO:0050779	RNA destabilization	0.000752	0.251	16.78
**47**	GO:0043506	regulation of JUN kinase activity	0.000789	0.258	9.67
**48**	GO:0030154	cell differentiation	0.000802	0.257	2.15

**49**	GO:0000288	nuclear-transcribed mRNA catabolic process, deadenylation-dependent decay	0.000909	0.285	43.49
**50**	GO:0030638	polyketide metabolic process	0.000909	0.279	43.49
**51**	GO:0044598	doxorubicin metabolic process	0.000909	0.274	43.49
**52**	GO:0044597	daunorubicin metabolic process	0.000909	0.269	43.49
**53**	GO:0045935	positive regulation of nucleobase-containing compound metabolic process	0.000919	0.267	2.12
**54**	GO:1901653	cellular response to peptide	0.000977	0.278	5.29

Table S3. Psoriatic patients' information and experimental techniques were applied to their sam ples. #PSO1: psoriatic patient code 1; RNA-seq, RNA-sequencing; IF, immunofluorescence staining.

Psoriatic patients' information and experiments applied to their samples							
**Patient's Code**	**Gender**	**Age**	**PASI score before initiating systemic therapy**	**PASI score at the time of sampling**	**Therapy**	**Punch biopsies body origin**	**Samples used for**
**#PSO1**	Male	48	17.2	0	Ustekinumab	Never-lesional: Buttocks<br>Resolved: Thigh	RNA-seq, IF staining, and Real-Time RT-PCR
**#PSO2**	Male	35	18.4	18.4	Methotrexate	Never-lesional: Thigh<br>Resolved: Thigh	IF staining
**#PSO3**	Male	30	24.4	13.6	Methotrexate	Never-lesional: Abdomen<br>Resolved: Abdomen	IF staining
**#PSO4**	Male	65	15.6	2	Infliximab+ Methotrexate	Never-lesional: Trunk<br>Resolved: Upper arm	IF staining
**#PSO5**	Female	39	17.1	0	Ustekinumab	Never-lesional: Lower back<br>Resolved: arm	RNA-seq and Real-Time RT-PCR
**#PSO6**	Male	38	17	0	Ustekinumab	Never-lesional: Buttocks<br>Resolved: Right leg	RNA-seq and Real-Time RT-PCR
**#PSO7**	Male	26	18	0	Ustekinumab	Never-lesional: Thigh<br>Resolved: Thigh	RNA-seq

Table S4. Real-Time RT-PCR TaqMan assay Probes.

**Number**	**Gene Symbol**	**Catalog No**	**Assay ID**
1	TET1	4331182	Hs04189344_g1
2	TET2	4331182	Hs00325999_m1
3	TET3	4331182	Hs00896441_m1
4	18s rRNA	4331182	Hs99999901_s1
